# Saccharomyces cerevisiae for abdominal pain and discomfort in irritable bowel syndrome patients

**DOI:** 10.12669/pjms.40.3.8349

**Published:** 2024

**Authors:** Mehreen Siyal, Zaigham Abbas, Muhammad Rafay Amir, Muhammad Ali Qadeer

**Affiliations:** 1 Mehreen Siyal, MBBS Department of Gastroenterology and Hepatology, Dr. Ziauddin Hospital, Clifton Campus, Karachi - Pakistan; 2 Zaigham Abbas, FCPS, FACG Department of Gastroenterology and Hepatology, Dr. Ziauddin Hospital, Clifton Campus, Karachi - Pakistan; 3 Muhammad Rafay Amir, MBBS Department of Gastroenterology and Hepatology, Dr. Ziauddin Hospital, Clifton Campus, Karachi - Pakistan; 4 Muhammad Ali Qadeer, MBBS Department of Gastroenterology and Hepatology, Dr. Ziauddin Hospital, Clifton Campus, Karachi - Pakistan

**Keywords:** Irritable bowel syndrome, Saccharomyces cerevisiae, Abdominal pain, Abdominal discomfort, Quality of life

## Abstract

**Background:**

Irritable Bowel Syndrome (IBS) leads to significant impairment of health-related quality of life, for the alleviation of which, the efficacy of available therapies is modest. Limited data is available on the role of Saccharomyces cerevisiae in treating patients with IBS.

**Methods:**

Thirty patients with IBS as per Rome-IV criteria, visiting our outpatient department from March 2021 to October 2021, were given capsule Saccharomyces cerevisiae 500 mg twice daily for four weeks. Evaluation for abdominal pain symptoms was done every week and the patient’s compliance was assessed. IBS Quality of Life (QOL) questionnaires were filled at baseline and after four weeks of treatment. The QOL and pain scales were adjusted to 0-100 for statistical analysis.

**Results:**

Seventeen patients (56.7%) were males. The age range was 21-72 years (mean ± SD: 39. 63 ± 14.32), out of which 18(60%) patients were 20-40 years old. Body Mass Index (BMI) ranged from 18-33 (25.33 ± 4.09), and 17 (56.67%) were overweight or obese. Sixteen patients had constipation predominant (53.3%), nine had diarrhea-predominant (30%), and five had mixed-type (16.7%) IBS. There was an improvement in the pain score from 63.81 at week 0 (W0) to 20.48 at the end of week 4 (W4) (p<0.001). An improvement was noted in all the eight categories of IBS QOL questionnaire, i.e., dysphoria (p<0.001), interference with activity (p<0.001), body image (p<0.001), health worry (p<0.001), food avoidance (p<0.001), social reaction (p<0.001), sexual function (p<0.001) and relationships (p<0.001). There was an overall improvement in QOL score from a mean of 24.68 at baseline to 58.09 at the end of the study duration (p<0.001). The improvement in the pain score showed a positive correlation with the improvement in quality of life (p<0.001).

**Conclusion:**

Treatment with Saccharomyces cerevisiae improved the pain and quality of life in patients with IBS and it appears to be a promising option for alleviating symptoms in these patients.

## INTRODUCTION

Irritable Bowel Syndrome (IBS) is a common functional gastrointestinal (GI) disorder characterized by abdominal pain or discomfort, associated with a change in bowel habits. It has been sub-typed according to the cardinal bowel habit as Irritable Bowel Syndrome Constipation predominant (IBS-C), Irritable Bowel Syndrome Diarrhoea predominant (IBS-D), Irritable Bowel Syndrome Mixed type (IBS-M), and Irritable Bowel Syndrome Un- subtyped (IBS-U).^1^ It has an estimated worldwide prevalence of 11.2%,^2^ accounting for 25% of gastroenterology outpatient consultations.^3^ It can lead to significant impairment of quality of life, social isolation, or stigmatization,^4^,^5^ decreased work productivity, increased healthcare, and societal costs.^6^-^8^

To date, the pathogenesis of IBS is not clear. Different mechanisms have been implicated such as altered gastrointestinal motility, visceral hypersensitivity, intestinal barrier disorder, dysfunction of the brain-gut axis, intestinal inflammation, and disruption of intestinal microflora.^9^,^10^ The gut microflora is of great interest when investigating IBS. Recent studies have shown dysbiosis of gut flora acting as a potential orchestrator of this disorder.^11^ There is a lack of satisfactory pharmacological treatments for the long-term management of IBS.^12^ Probiotics prove to be a promising option that may help to counterbalance the involved mechanisms as well as mitigate the symptoms.^13^

Saccharomyces cerevisiae is a yeast strain that may help in attenuating GI symptoms in IBS patients.^14^ It secretes saccharolytic enzymes that assist intestinal flora in the production of short-chain fatty acids and alcohols that regulate the small intestinal motility, thus having a possible role in patients with IBS.^15^ It also acts as a strong visceral analgesic and intestinal anti-inflammatory agent, indicating its beneficial effects in all subtypes of IBS.^14^ We aimed to assess the efficacy of Saccharomyces cerevisiae for symptomatic relief and improving the quality of life in patients with IBS.

## METHODS

This was a single-centre prospective observational study conducted at Dr. Ziauddin University Hospital, Clifton, Karachi Pakistan, from March 2021 to October 2021.

### Ethical Approval:

The study was approved by the Ethics Review Committee of Ziauddin University (Reference code: 2731020MSGE) and was also registered on clinicaltrails.gov having an ID: NCT05149599.

**Fig.1:**
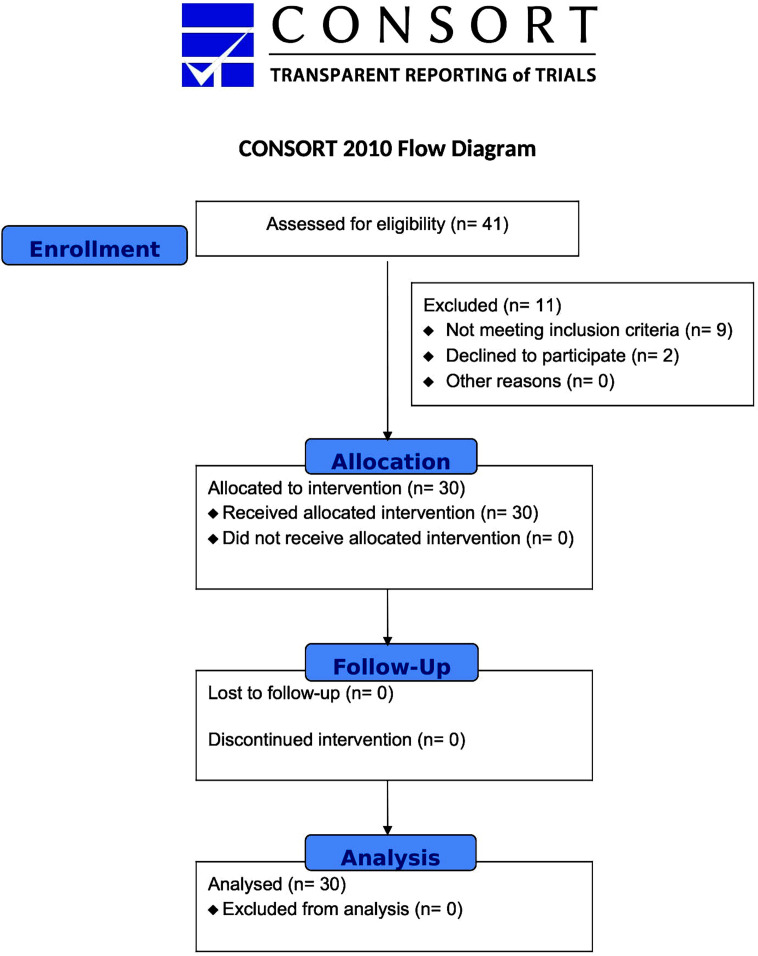


Thirty patients with IBS as per Rome-IV criteria, visiting our outpatient department were given capsule Saccharomyces cerevisiae 500 mg twice daily for four weeks. A written informed consent was taken from all the study participants. Previously validated IBS Quality of Life (QOL) questionnaires were filled, and severity of pain/discomfort was assessed at baseline using arbitrary grading from 0 (no symptoms) to seven (severe symptoms). Evaluation was done every week by phone for assessing pain/discomfort score and patient’s compliance. The patients were called for a formal outpatient visit after four weeks of initial treatment (Fig.1). The pain severity and QOL were reassessed and scales were adjusted to 0-100 for statistical analysis.

### Inclusion Criteria:

Male and female patients between 21 and 72 years of age, having confirmed IBS according to Rome IV criteria (newly diagnosed and previously non-responders to treatment), pain/discomfort score strictly above one and strictly below six, not hypersensitive to any of the ingredient of the drug, previous non-responders to conventional IBS treatment.

### Exclusion Criteria:

Patients with an organic intestinal disease (inflammatory bowel disease, celiac disease, lactose intolerance), patients on treatments likely to influence IBS (anti-depressants, laxatives, anti-muscarinic drugs, prokinetics, opioids, and narcotic analgesics), a recent history of taking antibiotics within four weeks of recruitment, patients with chronic alcoholism, vegetarian or vegan regimens, history of GI cancer in the past or at the time of recruitment, prior history of major abdominal surgery, severely immunocompromised and/or on immunosuppressants, severe systemic disease, major psychiatric illness, eating disorders such as anorexia or bulimia, documented food allergies, uncontrolled diabetes, marked thyroid dysfunction, pregnant females and patients who did not give written informed consent for getting recruited into the study.

The primary endpoint of the study was to determine the response of Saccharomyces cerevisiae for symptomatic improvement in patients with IBS through pain/discomfort score in seven days preceding the inclusion visit.^14^ Responders were defined as the patients who had an improvement of 50% of the weekly average intestinal pain/discomfort score compared with the baseline average score for at least two out of the four weeks of study duration.^16^

The secondary endpoint was to evaluate the response of saccharomyces cerevisiae for symptomatic improvement in patients with IBS through IBS-QOL questionnaire. IBS-QOL was evaluated using a validated 34-item IBS-QOL questionnaire (supplementary data, S1 Questionnaire), with each item scored on a 5-point Likert response scale (1=not at all, 2=slightly, 3=moderately, 4=quite a bit, and 5 =extremely or a great deal).^17^ The individual responses to the 34 items of the IBS-QOL questionnaire were summed and averaged for a total score and then transformed to a 0-100 scale for ease of interpretation, with higher scores indicating better IBS specific quality of life.

The transformation formula used for the IBS-QOL total and scale scores was: score = (the sum of the items-lowest possible score/possible raw score range) × 100. There were also eight subscale scores for the IBS-QOL (dysphoria, interference with activity, body image, health worry, food avoidance, social reaction, sexual function and relationships). Patients with an improvement in IBS - QOL overall score of ≥20 points from baseline were labelled as having improved QOL.^18^

### Statistical analysis:

Paired t-test was used to appraise the improvement in pain/discomfort score and the IBS-QOL from baseline. A p-value of less than 0.05 was considered significant. Statistical Package for Social Sciences (SPSS) version 23 was used for data analysis.

## RESULTS

A total of 30 patients with IBS diagnosed as per Rome IV criteria, either newly diagnosed or non-responders to the usual IBS treatment were included in this study. Seventeen (56.7%) patients were males. The age range was 21-72 years (mean ± SD:39.63 ± 14.32), out of which 18 (60%) patients were 20-40 years old. Twenty-one patients did not have any comorbid conditions, whereas three (10%) were hypertensive, two (6.7%) had concomitant dyslipidaemia, one (3.3%) was a known case of diabetes, one (3.3%) had a history of ischemic heart disease, one (3.3%) had prior chronic kidney disease and one (3.3%) had gastroesophageal reflux disease. The bodyweight of 14 (47%) patients ranged between 61-80 kgs. Seventeen (56.67%) patients had an overweight or obese Body Mass Index (BMI). Sixteen (53.33%) patients had IBS-C, nine (30%) had IBS-D, whereas five (16.67%) had IBS-M. The baseline demographics and scores of responders and non- responders have been summarized (Table-I).

**Table-I:** Baseline demographics and scores of responders and non-responders.

	Responders (n=26)	Non-responders (n=4)
***Gender***		
Male	15 (57.7%)	2 (50%)
Female	11 (42.3%)	2 (50%)
***Age***		
20-40 years	17 (65.4%)	1 (25%)
41-60 years	6 (23.1%)	3 (75%)
61-80 years	3 (11.5%)	0 (0%)
***Weight***		
40-60 kgs	7 (26.9%)	1 (25%)
61-80 kgs	13 (50%)	1 (25%)
81-90 kgs	5 (19.2%)	2 (50%)
Above 100 kgs	1 (3.8%)	0 (0%)
***BMI***		
Underweight	2 (7.7%)	1 (25%)
Healthy	10 (38.5%)	0 (0%)
Overweight	11 (42.3%)	3 (75%)
Obese	3 (11.5%)	0 (0%)
***IBS sub-type***		
Diarrhea-predominant	8 (30.8%)	1 (25%)
Constipation-predominant	14 (53.8%)	2 (50%)
Mixed-type	4 (15.4%)	1 (25%)
Treatment Naive	16 (61.5%)	2 (50%)
Treatment experienced	10 (38.5%)	2 (50%)
***Scores adjusted to a range 0-100***		
***Pain/discomfort score***		
W0	64.83±1.81	57.14±5.83
W1	32.97±1.73	46.43±3.57
W2	24.72±1.49	39.29±3.57
W3	19.23±1.36	39.29±3.57
W4	18.13±1.27	37.71±4.12
***Total IBS-QOL***		
W0	22.20±1.31	40.81±3.29
W4	58.40±2.37	56.07±3.86
***Dysphoria***		
W0	25.36±1.74	47.66±5.00
W4	62.50±3.29	60.16±3.69
***Interference with activity***		
W0	21.29±1.51	41.07±3.09
W4	57.28±2.85	52.68±3.38
***Body image***		
W0	19.95±2.02	35.94±5.34
W4	54.33±2.90	53.12±10.97
***Health worry***		
W0	22.76±1.64	43.75±8.59
W4	58.97±2.65	60.42±9.24
***Food avoidance***		
W0	26.60±1.96	39.58±7.12
W4	73.40±4.06	54.17±11.02
***Social reaction***		
W0	22.60±1.77	35.94±2.99
W4	57.69±3.47	59.37±4.03
***Sexual function***		
W0	11.54±2.39	37.50±5.10
W4	42.31±4.76	56.25±6.25
***Relationship***		
W0	20.51±2.37	35.42±6.25
W4	51.60±3.76	50.00±3.40

Values are n (%) or mean ± standard error of mean; W: Week.

**Fig.2:**
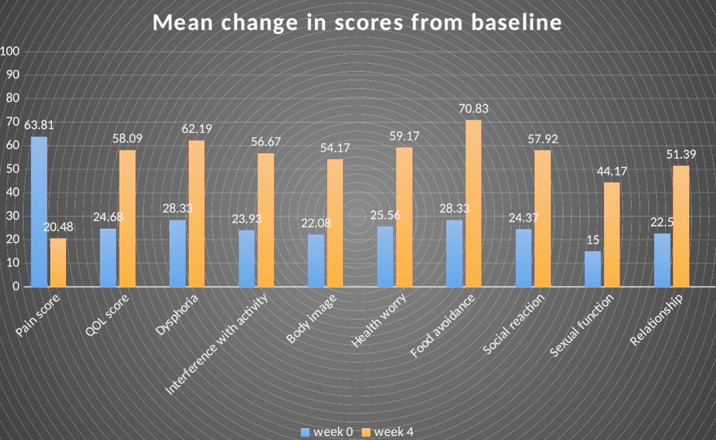
Mean change in pain score, QOL score and QOL subscales from baseline.

There was an overall improvement in pain/discomfort score from 63.81 at W0 to 20.48 at the end of W4. Twenty-six (86.67%) patients had a reduction of 50% for at least two out of the four weeks of the study period, and were labelled as responders, having an improvement in pain score from 64.83 at W0 to 18.13 at W4 (Fig.2).

There was a significant improvement in total IBS-QOL from 24.68 at baseline to 58.09 at the end of the study duration as shown in Fig.2. Similar to what was observed for pain scores, 26 (86.67%) patients had ≥ 20 points improvement in IBS-QOL and were labelled as responders, having improvement from 22.20 at W0 to 58.40 at W4. There was an improvement in all the eight subscales of IBS-QOL questionnaire (supplementary data, S2 Questionnaire), i.e., dysphoria (p<0.001), interference with activity (p<0.001), body image (p<0.001), health worry (p<0.001), food avoidance (p<0.001), social reaction (p<0.001), sexual function (p<0.001) and relationships (p<0.001) (Table-II). The improvement in the pain score showed a positive correlation with the improvement in IBS quality of life (p<0.001).

**Table-II:** Improvement in pain score, total Quality of Life (QOL) score and QOL subscales.

Improvement in scores	Mean±sem	95% confidence interval of the difference		Sig (2-tailed)

		LOWER	UPPER	
Pain score	43.33±2.51	38.19	48.48	<0.001*
Total QOL	33.41±1.71	29.92	36.89	<0.001*
Dysphoria	33.85±2.34	29.07	38.64	<0.001*
Interference with activity	32.73±2.21	28.21	37.26	<0.001*
Body image	32.08±1.96	28.08	36.09	<0.001*
Health worry	33.61±2.21	29.10	38.12	<0.001*
Food avoidance	42.50±3.42	35.50	49.50	<0.001*
Social reaction	33.54±2.19	29.06	38.02	<0.001*
Sexual function	29.17±2.57	23.92	34.41	<0.001*
Relationship	28.89±2.21	24.36	33.42	<0.001*

* Statistically significant values. QOL, quality of life, SEM: Standard Error of Mean.

## DISCUSSION

IBS is one of the most common functional GI disorders, imposing a substantial economic burden on patients and healthcare systems.^19^ It presents with divergent clinical symptoms, which make it difficult to treat. Various treatment options are available for alleviating symptoms of IBS, including laxatives, fibres, chloride channel activators, antispasmodics, antidepressants, serotonin antagonists, loperamide, 5-HT receptor antagonists, and rifaxamine. These treatment options focus on alleviating the symptoms of IBS, but are often unsatisfactory, warranting the need of effectual treatment for providing gratifying improvement in abdominal pain and quality of life.^20^ The role of probiotics in combating the symptoms of IBS by causing alterations in the intestinal flora, enhancing mucosal integrity, and inhibiting gut inflammation has been proposed by some trials, however, the jury is still out regarding validating their efficacy for treating IBS.^21^

The yeast strain Saccharomyces cerevisiae has been found to produce a significant reduction in abdominal pain through local activation of the Peroxisome Proliferator-Activated Receptor alpha (PPARα) in addition to the modification of gut flora.^14^ It also produces short-chain fatty acids and alcohols, which results in decreased luminal pH and the production of bactericidal proteins.^15^ Saccharomyces boulardii is another yeast strain that has been used in patients with IBS, but previous studies have suggested that its role in ameliorating the symptoms of IBS lacks appropriate strength of evidence and is modest.^22^

Many of our patients had IBS-C (53.3%). However, Saccharomyces cerevisiae neither worsened the stool frequency and consistency in IBS-D nor did it bring any periodic fluctuation in the symptoms of diarrhoea and constipation in IBS-M, proving that it helps in the modulation of gut motility in all sub-types of IBS by acting on PPARα receptors. These findings were like a previous study done by Gayathri et al.^23^

Pain/discomfort score is an important tool while evaluating response to treatment in a patient with IBS.^14^ Our study patients reported a consequential reduction in this score, which was assessed through phone calls every week and physical presence at the end of the study period. IBS-QOL questionnaire is a 34-item containing questionnaire which evaluates various aspects of an IBS patient’s quality of life. In our study, patients got an improvement in all the eight sub-scales of IBS-QOL, each having a significant p-value of <0.001. A captivating finding was the fact that four (13.3%) patients who were categorized as non-responders in the context of pain/discomfort score were found to have an improvement in IBS-QOL of <20 points, emphasizing a possible link between the two scoring systems. Moreover, a positive correlation was found between the improvement in pain/ discomfort score and IBS-QOL (p<0.001).

Two adverse events were reported in our study. One patient experienced nausea, while another patient encountered epigastric discomfort during the initial days of the study period. However, these events were transient, and trivial, and did not lead to any dropout in this study. This finding proclaimed that Saccharomyces cerevisiae was well tolerated by most of our study subjects.

### Strength of the study:

Firstly, two scoring systems were used, focusing not only on improvement in pain but also on quality of life in patients with IBS. Second, patients were closely followed through phone calls every week to keep a check on patient compliance and to know any adverse effects of the medication. Third, there was no dropout. All the patients completed the study duration of four weeks. Fourth, most of the studies conducted so far regarding probiotics in IBS mostly used them as add-on medications in addition to the conventional drugs used to treat IBS. But in this study, Saccharomyces cerevisiae was given as a single treatment agent. Fifth, previous studies on Saccharomyces cerevisiae were conducted mostly in patients with IBS-C, whereas in our study patients with IBS-D and IBS-M were also included. Sixth, drug response was seen both in treatment naive as well as in patients who did not respond to the conventional drug therapies, thus emphasizing the fact that this drug appears to be a satisfactory option in patients not responding to prevailing IBS therapies.

### Limitations:

The sample size was small because the drug sample was available for a limited number of patients. The Control group was not present, which could have further validated our findings. The role of this drug was not compared with other medications used in IBS. The study period was just one month and tests at the molecular level were not conducted.

## CONCLUSION

Our study concludes that Saccharomyces cerevisiae can be a good treatment option, for assuaging abdominal pain and improving the quality of life in patients with IBS.

### Study Highlights:

***What is known:***

- Gut dysbiosis plays an important role in the pathogenesis of IBS.
- The role of probiotics in treating patients with IBS is not well-established.

***What is new:***

- Yeast strain Saccharomyces cerevisiae is effective as a single treatment option for improving abdominal pain and quality of life in patients with IBS.

### Authors Contribution:

**MS** and **ZA:** Data collection, analysis, interviewing the patients, manuscript writing.

**MRA:** Helped with data entry.

**MAQ:** Helped in data collection. All authors reviewed the manuscript and approved the final version.

***Zaigham Abbas:*** Takes the responsibility for the integrity of the study.
